# Breath in the technoscientific imaginary

**DOI:** 10.1136/medhum-2016-010908

**Published:** 2016-08-19

**Authors:** Arthur Rose

**Keywords:** Respiratory medicine, Film

## Abstract

Breath has a realist function in most artistic media. It serves to remind the reader, the viewer or the spectator of the exigencies of the body. In science fiction (SF) literature and films, breath is often a plot device for human encounters with otherness, either with alien peoples, who may not breathe oxygen, or environments, where there may not be oxygen to breathe. But while there is a technoscientific quality to breath in SF, especially in its attention to physiological systems, concentrating on the technoscientific threatens to occlude other, more affective aspects raised by the literature. In order to supplement the tendency to read SF as a succession of technoscientific accounts of bodily experience, this paper recalls how SF texts draw attention to the affective, non-scientific qualities of breath, both as a metonym for life and as a metaphor for anticipation. Through an engagement with diverse examples from SF literature and films, this article considers the tension between technoscientific and affective responses to breath in order to demonstrate breath's co-determinacy in SF's blending of scientific and artistic discourses.

## Introduction

This article considers how breath's affective qualities may interrogate implicit claims to medical realism in science fiction (SF). I argue that SF is not constrained to impressions of technoscientific plausibility and scientific modelling when responding to the affective experience of breathing.^1^ SF in fact engages with questions of affective breathing in provocatively non-scientific ways, even when articulated through apparently scientific discourse. The result is a tension ‘between a technoscientific imaginary that disenchants [breath], and a sense that human experience will always be sublimated, because it is embodied, situated, partial, and fragmentary’.^1^ The breathing experience of SF novels or films always exceeds, in its affective qualities, its bare description as scientific process. Rethinking the affective qualities of breath in SF as they efface respiratory medical discourse (*Fahrenheit 451*), resist technical appropriation (*2001: A Space Odyssey*) or recall affective experiences often forgotten in the scientific languages of processes (*The Abyss*) can, I suggest, provide the basis for a more useful dialogue between affective and technoscientific discourses.

## The Cheyne-Stokes respiration of civilisation

In their February 1951 issue, *Galaxy Science Fiction* published a novella by Ray Bradbury.^2^ *The Fireman* would subsequently be expanded into the most successful novel of Bradbury's career, *Fahrenheit 451* (1953).^3^ The novel, named for the temperature at which paper burns, significantly embellishes on the world of the novella, a world in which, to facilitate the censorship of books, ‘firemen’ have been retooled to set fires rather than douse them. Amidst his elaborations, however, Bradbury omits one significant passage. Montag, the hero of both *The Fireman* and *Fahrenheit 451*, convinces his wife, Mildred, to spend an afternoon reading a pile of banned books he has secreted away from various burnings. When she challenges him to explain why he wants her to read the books, at risk of personal ruin, he tells her to ‘listen’ (p. 24;^2^ p. 74).^3^ In *Fahrenheit 451*, this is followed by a third-person description of bombers crossing the sky, her listening experience captured by a series of indirectly discursive sonic metaphors: ‘gasping, murmuring, whistling like an immense, invisible fan, circling in emptiness’ (p. 74).^3^ In *The Fireman*, ‘she listens’ to the bombers crossing the sky, ‘quick gasps in the heavens, as if a running giant had drawn his breath’, which precipitates a meditation on the disjunction between the ever-present sounds of the bombers and their visual absence: ‘they had heard those jet sounds and seen nothing, until, like the tick of a clock or a time-bomb, it had come to be unnoticed, for it was the sound of today, the Cheyne-Stokes respiration of civilization’ p. 24).^2^ In both texts, the incursion of the bombers will provoke Montag to an outburst about the society's continuous state of war and gives rise to his suggestion that reading might lead to a personal liberation, at least, from ‘the cave’ (Bradbury's allusion to Plato's ‘Allegory of the Cave’) (p. 74).^3^ But Bradbury's medical metaphor, ‘the Cheyne-Stokes respiration of civilization’ and its omission from *Fahrenheit 451* provide me with the starting point for thinking about the role of breath in the ‘technoscientific imaginary’.

‘The Cheyne-Stokes respiration of civilization’ conceives the disembodied jet sounds as a pathological form of breathing. Cheyne-Stokes respiration is named after two physicians: William Stokes and John Cheyne. In Stokes's *The Diseases of the Heart and Aorta* (1854), there is an extensive gloss on John Cheyne's 1818 observation of the breathing irregularities associated with damage to respiratory centres and chronic heart failures.^4^ Cheyne-Stokes respiration is characterised by a breathing cycle with three distinct phases. First, the breath gradually increases its depth and rapidity; then, it decreases this rapidity, eventually resulting in an apnoea or brief cessation. Following Bradbury's metaphor, civilisation breathes. That breath is pathologically irregular and characterised by speed-ups, slow-downs and crisis-stops. Although it might equally describe today's reliance on crash capitalism, ‘the sound of today’ for Bradbury, with his interest in Cold-War politics, is the ever-present, unseen war that disrupts the apparent stability of his dystopia and eventually collapses its illusory peace. By using the breathing cycle as the metaphor's source, Bradbury links the wider sociopolitical conditions of his anaesthetised society to more intimate markers of personal unhappiness (Mildred's dependency on sleeping medication, Montag's inability to remember a time when he was happy). The metaphor is not simply symbolic of connections between politics, health and personal life; it also alerts the readers to an intermittent motif of breath.

When Montag takes one of his illegal books to an acquaintance, he, like the ‘giant’ mentioned above, must stand ‘catching his breath’ after running away from an angry mob (p. 28).^2^ Catching a breath, holding a breath, increasing a breathing rate: Bradbury uses these to gesture to the ways various bodies exhibit their concern at the state's ‘breathing problems’. This problem is not simply a breathing pathology in the rhythm of the state; breath is in itself a form of biopolitical control. Montag realises, as he flees the Mechanical Hound that tracks him, that ‘the particles of his breathing might remain in an electronically detectable invisible cloud for hours after he had passed on’ (p. 50).^2^ When, in the last pages of the novella, Montag's city is destroyed, the force of the explosion will leave the rebel group he has joined, a group who seek to preserve the books by each keeping a single page, chapter or text in their heads, ‘gasping like fish’ (p. 60).^2^ This immanent destruction of civilisation will leave them temporary inheritors of the Cheyne-Stokes respiration, which gradually gives way to a new normality: ‘You could hear them breathing fast, then slower, then with the slowness of normality’ (p. 60).^2^ Even the ending of *The Fireman*, juxtaposing quotes from the books of Ecclesiastes and Job, *Hamlet* and ‘Ben Bolt’ with the immanent declaration ‘Montag felt fine’, implies, in its emphasis on the normality of transience and mortality, a new awareness of the need for slow breathing.

*Fahrenheit 451* retains traces of this breath motif, but with some critical differences. The rebels are also heard ‘breathing fast, then slower, then slow…’, but ‘the slowness of normality’ has been replaced with an ellipsis (p. 155).^3^ The rebels are not returning to normal; they are performing a strange form of Cheynes-Stokes breathing (fast, slow, cease). The implications of this relation between ‘death’ and ‘breath’ are evident early in the novel, when Montag returns home for the first time. The room he shares with Mildred is described as ‘cold but nonetheless he felt he could not breathe […] with the feeling of a man who will die in the next hour for lack of air, he felt his way toward his open, separate, and therefore cold bed’ (p. 19).^3^ Mildred herself is introduced as someone whose only signs of life are ‘the breath coming out of the nostrils’ (p. 20).^3^ In each instance, breath is immanent to the body that breathes, rather than normative and prescriptive: unlike *The Fireman*, *Fahrenheit 451* does not make the metaphysical connection between the pathologies of individual breathing patterns and the collapse of civilisation explicit. The ‘Cheyne-Stokes respiration of civilization’ is performed, rather than referenced.

Bradbury's decision to eliminate direct allusions to breath science from *Fahrenheit 451* makes aesthetic sense, in that it replaces ‘telling’ with ‘showing’. In *Fahrenheit 451*'s version of the ‘Cheyne-Stokes’ passage, the loss of a mediating ‘she listens’ allows for a more subtle indirect discourse, which parallels the sky's emptiness with Mildred's response: rather than her outburst in defence of her radio and television programmes detailed in *The Fireman*, she simply ‘snatched the phone’ when it rings in *Fahrenheit 451*, the quicker to return to her unhappy, cloistered existence (p. 74).^3^ More generally, the novel integrates ‘Cheyne-Stokes’ into its form, rather explicitly naming it as a concept. Where *The Fireman* links breathing pathology to civilisation explicitly, *Fahrenheit 451* performs these irregularities as a series of structural arrhythmias in the actions of the characters and the expectations of their society. Bradbury genders this arrhythmia in his treatment of Mildred, the hysterical exemplar of her civilisation's ‘Cheyne-Stokes’: she seeks breathlessness simultaneously in high speed driving (which ‘tore the breath from [Montag's] mouth’), her ‘programmes’ (which she sings along to ‘under her breath’) and in death (her attempted suicide).^3^ By contrast, Montag's stable, if perplexed, progress from his ‘pleasure to burn’ to his acceptance that ‘to everything there is a season’ orients itself towards a more ‘mindful’ pattern for everyday life. Mildred's instability, paired with Montag's comparable stability, points to Bradbury's problematic gender politics, since he seems to perpetuate particularly nineteenth-century scientific corollaries between pathological breath conditions, hysteria and the female subject. But the way in which he integrates a breathing condition into the form of the novel also opens up an alternative way of reading the role for breath in SF, a way that exceeds or disrupts description within the technoscientific imaginary.

## The technoscientific imaginary

In the ‘technoscientific imaginary’, or ‘the culturally-embedded imagining of futures enabled by technoscientific innovation’, breath risks being understood simply as a scientific process, rather than anything that disrupts scientific discourse.^5^ Breath, as object fact, yields itself to bald, empirical description. The metaphysical problem of breath is easily ‘solved’ by scientific process, as an answer to the empirical question asked of the work, namely ‘how do the characters or personae breathe?’ By no means a simple question, this ‘how’ encompasses the chemistry of what is breathed, its presence or absence from a particular environment and the processes by which it is inhaled or exhaled. Its bio-literary expediency is resolved by discoveries in the field of scientific concern.

The problem facing a technoscientifc aesthetic, particularly in its relationship with medical science and emergent technologies, is its presentation of technoscientific possibilities that themselves take as given the translation of certain scientific ideologies into scientific certainty. This manifests, in the case of breath, as a scientific certainty whose predicates might be explored but whose quiddity is not to be interrogated. Breath, the scientific process, always threatens to obscure breath, the cultural network. A corrective, proposed by Peter Adey, is what Marijn Nieuwenhuis has called Adey's

return to a revised and revived, pre-Socratic understanding of the element of air. Such an ‘anterior’ position enables Adey to decline temptations to reduce air to ‘geophysicalist epistemologies and ontologies’ and allows him to construct theorizations of air as a ‘backgrounding condition, structure, or “force”, but also and just as crucially, a method of affinitive listening’. (p. 90)^6^

By returning, at least conceptually, to cultural understandings of breath that are not underpinned by modern medical science, we open ourselves to alternative, perhaps conflicting, forms of understanding and articulating breath. For all its resemblance to such movements, this conceptual rehabilitation of prior models is not the correlative of either retrofuturism or steampunk: it is neither a ‘detached sensibility that mourns the lost belief in progress’ nor retrofit valorisation of ‘a mode of productive labor based on work with materials’ (pp. 249; 250).^7^ Rather, it responds to the scientific facticity of breath (exemplified by, for instance, ‘the Cheynes-Stokes respiration’ of *The Fireman*) with the possibility that a scientific definition might be but one way among many when responding to a particular experience that might be better ‘shown’ than ‘told’.

A further rationale for opening up ‘alternative discourses’ emerges when we note that breath, itself, presents a challenge to scientific realism. Conceptually, breath may be understood as a series of binary opposites entangled by process. Interior meets exterior as our lungs take in or give out air. Conscious and autonomic functions blur as we either concentrate on controlling, holding or releasing our breath or concede control to the regulation of our bodies. Cartesian distinctions between mind and body blur in the physiological and the spiritual understandings of breath, as we breathe either to modulate mood or to pace meditation. Even my use of ‘we’ is only possible because I assume that you and I share the biological-spiritual imperative *to breathe*. Breath transgresses, in its processes, the rigorous divisions of its parts. This transgression is detectable as an echo of scientific discourse in SF literature such as *The Fireman* and *Fahrenheit 451*; in SF film it emerges in a preference for affective sounds over scientific images.

## Breath and film

Davina Quinlivan devotes a page of *The Place of Breath in Cinema* (2012) to the ‘detached and scientific imagining of the human body’ found in Paul Thomas Anderson's *Magnolia* (1999) (p. 8).^8^ The need to address the acoustic quality of breath contributes to her decision not to explore the ‘technoscientific’ in greater detail. ‘Despite the visual impact of Anderson's microscopic images of respiration, they ultimately offer limited scope for reflection beyond the representational qualities of such images’ (p. 8).^8^ Quinlivan's meditation on Anderson's respiratory montage, where a cancerous cell is tracked in Earl Partridge's body, demonstrates the importance of thinking about breath's temporalities, rather than its scientific process. But Quinlivan explicitly links breath's acoustic qualities to SF in her discussion of Darth Vader from George Lucas's *Star Wars* (1977): ‘it is the strange “whooshing” sound of breathing associated with the Darth Vader character that is at the core of this iconic film’ (p. 5).^8^ ^9^

For Quinlivan, breath suggests ‘a mode of duality between the material and the incorporeal’ and emphasises ‘the particular unsettling of boundaries between vision and the unseen’ to create ‘an intermediary internal sound between conscious expression and involuntary, unintentional corporeality’ (pp. 3; 136).^8^ The result is an ‘(im)material’, ‘(in)visible’ ‘filmic presence’ (p. 3).^8^ This presence has a technoscientific explanation, ‘the sound of Darth Vader's breathing functions as a prominent reminder of his physical weakness’. ‘Yet, in spite of this knowledge, the particular feelings of discomfort that may be experienced by the viewer as a result of hearing Darth Vader's continuous, stifled breath contained within the mask remain difficult to explain’ (p. 5).^8^ Quinlivan proposes, by way of explanation for this discomfort, the emphasis on Vader's ‘living, suffering human body which underscores the issue of mortality in the film’ (p. 6).^8^ While *Star Wars* provides an excellent diegetic example for breath's (in)visibility, Quinlivan argues that the reliance on thinking breath as something that is *heard* allows breath's (im)materiality to be understood as something seen or felt.

Given the complexity of her analysis, it is unfortunate that Quinlivan, when looking at SF more generally, claims the genre has a thematic preoccupation with scientific breathing at the expense of more complex affective engagements, which she finds ‘much more bound up’ in horror films. In this regard, Quinlivan regards *Star Wars* as exceptional in its exploration of breath's affect. Generally, ‘SF cinema is preoccupied with oxygen’ (p. 24),^8^ ‘particularly through its thematic reference to the air-lock or lack of oxygen’ (p. 23).^8^ The recurrence of the airlock trope implies a technoscientific analysis of the need to breathe within the structures of plot and narrative. The character, locked out of ‘breathable space’, desires to re-enter this space and makes a number of attempts to do so. The plot turns on this character's inability to breathe. Whatever other fantastical postulates, the scientific economy of breath keeps characters bonded to the empirical process of breath, with her notion of ‘filmic presence’ conspicuously absent. The implication is that, for Quinlivan, the preoccupation that SF characters share over breathing is distinctly technoscientific. Stanley Kubrick's *2001: A Space Odyssey* (1968) provides Quinlivan with one such example, namely the scene where the computer, HAL 9000, locks the astronaut, Dave, out of the ship.^10^

Focusing on this moment, however, ignores the more compelling instance of breath as acoustic supplement to the film's visuals, particularly as it differentiates between respirable space within the astronaut's suit and irrespirable outer space. Breath also functions as a rhythmic counterpoint to HAL's melodious rendition of 19th century music-hall song ‘Daisy’ as Dave deactivates him. It also marks the stages of Dave's life, given in the succession of rooms that he visits. David W. Patterson notes that this

*musica humana* becomes a signature ‘theme’ at those moments when humankind's identity is asserted in sharpest contradistinction to other elements in the environment, comprising specifically confrontations with nature (as a lone astronaut floats in the endlessness of space), technology (as the conflict between human and machine culminates in HAL's termination) and the alien or ‘other’ (as an astronaut who has ostensibly traversed space and time lands inexplicably in a suite of rooms). (p. 462)^11^

Patterson's attention to Kubrick's musical treatment of breathing sounds (the *musica humana*) demonstrates that, far from being a scientific feature of the film, breath becomes a metonym for a peculiarly unscientific aspect of the human (epitomised by Dave), expressed in its encounters with the non-human (represented by HAL 9000). *2001* provides a stimulating contrast between the inhuman technoscientific and its human counterpart. But the consequence of this dialectic is to re-establish a dichotomy in which breath is understood through either the technoscientific (as non-human) or the human (as extra-technoscientific). To consider this dichotomy in a more useful way, where the technoscientific and the extra-technoscientific may be fused rather than contrasted and thus develop the haptic aesthetic that Quinlivan finds in the more experimental cinema of Atom Egoyan, David Cronenberg and Lars von Trier, I turn to a final visual-acoustic example from the SF film: James Cameron's *The Abyss* (1989).^12^

## The Abyss

*The Abyss* most obviously coordinates a relationship between technoscientific breath and its extra-technoscientific corollary when Ed Harris' Bud Brigman prepares to descend the Cayman Trough to disarm a Trident missile. Bud works on an experimental underwater oil rig, which is appropriated by the US Navy to retrieve the Trident missiles from a sunken US nuclear submarine. His estranged wife, Lindsey Brigman, the designer of the rig, joins the expedition, as do a number of US Navy Seals, the crew of the rig and the pet rat of one of the crewmen. A storm severs their connection to the surface, even as one of the Navy Seals, Hiram Coffey, begins to suffer paranoia induced by high-pressure nervous syndrome. When it comes to light that the sinking of the submarine was caused by the presence of non-terrestrial intelligences (NTIs), Coffey attempts to blow up the NTIs with one of the Trident missiles. A submarine battle between the Brigmans and Coffey sees Coffey die in the trench, but not before the missile sinks down to the NTIs' city. Bud must then descend the trench to disarm the missile. Sea pressure at that level renders gaseous oxygen unbreathable. The film's technoscientific solution to this dilemma is an oxygenated fluorocarbon emulsion, which will permit Bud to ‘breathe’ for a limited period of time. Changing the ‘what’ (oxygenated emulsion) and the ‘how’ (liquid respiration) of Bud's breathing process resolves the problems posed by the alteration in environment. Fantasy becomes reality as the details of how Bud is to breathe are explored in a series of small, yet important scenes.

Our introduction to liquid breathing occurs when the Navy Seals first arrive on the oil rig. Their dive master explains the experimental dive equipment to the owner of the rat. To illustrate his explanation, he puts the rat into a cage, which he then submerges in the emulsion. This sequence, cut from British versions of the film because of perceived cruelty to animals, anticipates Bud's immersion in the same liquid. Unlike those scenes filmed with Harris, however, the rat really breathed the fluorocarbon mixture: ‘what you see is a rat breathing a liquid. There are no tricks, no special effects of any kind’ (p. 95).^13^ The technoscientific ‘innovation’ of liquid breathing had already been realised in 1989 by Johannes Kylstra. Kylstra, whom Cameron consulted before the film in 1987, published his findings on the feasibility for humans to breathe through oxygenated fluorocarbons in 1977, and his animal test studies date back to 1962.^14^ James Cameron recalls meeting Francis Falejczyk, the diver Kylstra worked with, when he was 16 or 17 (between 1969 and 1971). The meeting led Cameron to write a short story, titled ‘The Abyss’, set in an underwater laboratory and premised on the use of liquid breathing.^13^ Even at this time, he knew from Falejczyk that Kylstra had concluded that, while divers might use oxygenated fluorocarbons at rest, the increased levels of oxygen when at work would make the suitable oxygenated fluorocarbons prohibitively toxic. Falejczyk himself developed pneumonia after the experiment. Certainly, while forms of liquid breathing are used in neonatal care today, there is no successful counterpart in adults, where relative lung space does not allow for sufficient absorption from full liquid immersion. Liquid breathing, a pivotal feature of *The Abyss*, provides a provocative example of the technoscientific imaginary's role in considering oxygenated fluorocarbons as an emerging or future medical technology.

The scientific limit for these questions is framed, then, as the limits to which such technologies may be pushed. The film, by contrast, suggests that the discourses around emergent technologies are usefully opened up by considering their elective affinities with other, more literary, narratives. The clearest example is to be found in parallel situations involving the rat, Bud and Lindsey. The rat's immersion in the fluorocarbons anticipates Bud's immersion, implicitly linking the ‘experimental’ breathing apparatus to the ethics of Kylstra's animal/human testing protocols. But both experiences also parallel Lindsey's drowning. Caught in a damaged submarine with only one air supply after dispatching the psychopathic Coffey, Bud and Lindsey are faced with the dilemma that they cannot both return to the oil rig. Lindsey's implausible solution is to drown herself. The low temperature of the water will slow her possible brain damage, providing Bud with the time necessary to return her to the rig and revive her. This ‘resurrection narrative’ has an established place in literary and theological traditions, but it also mimics the *change in state* of both the rat and Bud. All three ‘drown’ in the conventional sense of breathing in liquid but survive the process because their environment has been transformed. In each situation, the environment changes from air to water, their breathing changes as a result and the film depicts both changes as traumatic.

In all three situations, a scientific or personal explanation is used to normalise the changes. The divemaster attempts to ameliorate the trauma in the rat and, later, in Bud by repeating that their violent responses to the change in environment (from air to liquid) is either ‘completely normal’ or follows ‘a normal adjustment period’, appealing to personal experience, ‘I've done this myself’ and overlaying the violence of the visual transformation with a measured scientific narrative.^12^ As the camera focuses on the rat, during her immersion in the emulsion, the divemaster details what is happening:

He takes the fluid into his lungs. He takes the fluid into his lungs. There he goes. Now there's a bit of anxiety here. Now he's starting to relax. He's fine. See his chest moving. Getting plenty of oxygen […] See, the fluid's harder to push in and out than air. A little more work to breathe. He's going to be fine. He's digging it.^12^

The repetitions included in the description, combined with the diminution of the anxiety and the rationalisation of apparent difficulties, lend the divemaster's voiceover the appearance of objective scientific observation. The divemaster's objectivity may be less sympathetically understood as a lack of care, already marked in his refusal to change his pronominal referent for the rat from ‘he’ to ‘she’ despite repeated prompting. Rather than easing the process, the divemaster's explanation attempts to efface the traumatic transformation by detailing the steps as a straightforward progression. Resistance is recast as the simple struggles of a creature unhabituated to this process. However, neither tone nor stages can entirely erase the visual impact of the trapped rat's struggles as she clasps the cage before submitting to the new breathing protocol. As the crewman owner of the rat remarks, ‘she's doing it, she ain't digging it’.^12^ The scientific ‘normality’ of liquid breathing, assumed by the divemaster, is called into question by the sheer violence of Cameron's presentation of the transformation process.

This presentation is reiterated in the respective liquid immersions of Lindsey (her drowning) and Bud (his mask is filled with the emulsion). In each scene, the character repeats the rat's clasp. The camera focuses on Lindsey's hand during her drowning, as it phases from clutching Bud's helmet to extending stiffly in the visual equivalent of a death rattle, before relaxing out of the frame. The hand's gesticulation functions as a synecdoche for Lindsey's transformation from living, breathing body to liquid-breathing corpse. It is the visual correlative to the inherent resistance to breathing liquid. It recalls the rat's clasp, while anticipating Bud's own attempts to grab hold of something as his body resists the transubstantiation of air into liquid.

The aforementioned normative function of the scientific explanation is to distract precisely this attention to the traumatic grip. Lindsey gives a scientific explanation for drowning herself: ‘I can be revived after ten, fifteen minutes’.^12^ When Bud transitions from breathing air to breathing liquid, the divemaster insists on the normality of the process: ‘Relax […] Don't hold your breath, take it in […] It's perfectly normal. We all breathe liquid for nine months, Bud, your body will remember’.^12^ The actual scientific content of these explanations is less important than the dynamic they set up; in each instance, the traumatic content of the lived experience of drowning is (inadequately) explained away by discourses that seek a rational, technoscientific solution to the problem presented. Breathing, explained as a technoscientific function, does not adequately address affective responses to the presentation of liquid breathing. The rat and Bud may anticipate scientific advances in liquid ventilation when they begin to breathe the emulsion, but focussing on this novelty obscures the violence of the transition from one state of breathing to another, recalled in Fulke Greville's ‘Caelica 83’: ‘You that seek what life is in death,/Now find it air that once was breath’.^15^ The transition from life to death is understood through the metaphor of breath transformed into air, since to understand life through death, as the workings of bodies, is to understand breath as simply, technoscientifically, a matter of air. Greville's associative chiasmus (his first term, ‘life’, corresponds to his fourth, ‘breath’, while his second term, ‘death’, corresponds to his third, ‘air’) troubles any easy logic of transformation: what life becomes in death (a corpse) can be correlated to ‘mere’ air only because the traumatic transitions of breath no longer obtain. Adapting Greville's insights to *The Abyss*, when breath becomes liquid, we find the transitionary violence from life into death experienced by the rat, Lindsey and Bud, a violence whose ontological force is spent in a technoscientific imaginary where the interchangeability of liquid and air are, as the divemaster might say, ‘perfectly normal’.^12^

## Concluding remarks

My article addresses breath in SF by interrogating its appearance in the technoscientific imaginary. There is a possible tautology in scientific responses to breath, since breath may so easily be understood in wholly scientific terms. This results in a potential circularity in analyses that addresses the question of breathing in SF exclusively through the technoscientific imaginary. If writers are primarily concerned with realist portrayals what their characters breathe, even technoscientific reconstructions of how these characters breathe will maintain the ontological inference that they either breathe or do not. In fact, far from providing breath with a technoscientific support, or even advancing the science of breathing in the technoscientific imaginary, SF draws on breath as a ready correlative to embodied experience. It uses breath, in its embodied sense, to blend into the technoscientific imaginary, an extra-scientific affect. Breath is SF's corrective to the technoscientific imaginary, refining articulated through carefully staged scientific explanations and the affective conditions of haptic transformations and transubstantiations.
